# Ebola Conquers West Africa — More to Come?

**DOI:** 10.1016/j.ebiom.2014.10.004

**Published:** 2014-10-13

**Authors:** Peter Halfmann, Gabriele Neumann, Heinz Feldmann, Yoshihiro Kawaoka

**Affiliations:** a Department of Pathobiological Sciences, School of Veterinary Medicine, Influenza Research Institute, University of Wisconsin, Madison, WI 53711, United States; b Laboratory of Virology, Division of Intramural Research, National Institutes of Health, Hamilton, MT 59840, United States; c Department of Microbiology and Immunology, Division of Virology, International Research Center for Infectious Diseases, Institute of Medical Science, University of Tokyo, Tokyo, Japan

In 1976, the world's attention was caught by two almost simultaneous outbreaks of a new and then a mysterious virus named Ebola (Fig. 1) in the Sudan and what was then Zaire, now known as the Democratic Republic of Congo. Since then, sporadic outbreaks caused by Ebola virus have resulted in approximately 2387 cases and 1590 deaths (http://www.who.int/mediacentre/factsheets/fs103/en/). This year, Ebola virus returned to the center stage as the Western African countries of Guinea, Liberia, and Sierra Leone are facing an unprecedented and uncontrolled outbreak caused by a new *Zaire ebolavirus* strain that has been linked by epidemiological evidence to a potential “patient zero” who succumbed to infection in December, 2013 (Baize et al., 2014). The number of cases in this outbreak has surpassed that for all previously reported Ebola virus outbreaks combined. As of September 18, 2014, there have been more than 5347 cases with 2630 deaths; these numbers include 318 infected healthcare workers, half of whom have died (http://www.cdc.gov/vhf/ebola/outbreaks/guinea/index.html). In past outbreaks, the case fatality rates have varied from 25% to 90% (http://www.who.int/mediacentre/factsheets/fs103/en/); the case fatality rate of the current outbreak is 53% (http://www.cdc.gov/vhf/ebola/outbreaks/guinea/index.html.). However, due to limited access to Ebola treatment units and the stigma associated with this disease, many infections may have gone unreported, so the true number of infected people is most likely much higher, and the true case fatality rate will not be known until the outbreak is over.

The early symptoms of Ebola virus hemorrhagic fever disease are non-specific: fever, malaise, muscle pain, diarrhea, and vomiting. Eventually, recognizable hemorrhagic symptoms are evident including vomiting of blood, nosebleeds, and a characteristic rash, late in the course of the disease, in roughly half of those infected (http://www.who.int/mediacentre/factsheets/fs103/en/). Transmission occurs through direct contact with virus-contaminated body fluids (blood, secretions, or other bodily fluids), materials such as bedding contaminated with these fluids, through the handling and preparation of contaminated food such as bush meat, or through direct or indirect contact with infected bats, a potential animal reservoir for Ebola virus (http://www.who.int/mediacentre/factsheets/fs103/en/, n.d, Leroy et al., 2009).

Nearly four decades after the discovery of Ebola virus, we still lack licensed vaccines and antiviral therapeutics to treat Ebola virus-infected patients. However, considerable progress has been made in more recent years, and the current outbreak has fuelled major efforts to evaluate candidate therapeutics in clinical trials.

Supportive care that includes largely oral rehydration with solutions containing electrolytes is the typical course of treatment for Ebola-infected patients. Currently, several experimental Ebola virus vaccines confer varying degrees of protective efficacy in animal models. An ideal Ebola virus vaccine would elicit strong and protective responses after a single immunization and have therapeutic benefits. One of the most extensively tested Ebola virus vaccine platforms is based on a replication-competent vesicular stomatitis virus (VSV) expressing Ebola virus glycoprotein(s) (the major viral immunogen). A single dose of 10^7^ recombinant VSV particles has been shown to be effective against Ebola, and its close relative Marburg virus, in prophylactic and post-exposure situations in nonhuman primate models (review; Marzi et al., 2011). The use of replication-competent vaccine viruses always raises safety concerns; however, such concerns will be addressed in current and future clinical trials.

Another vaccine platform is based on a replication-defective, chimpanzee adenovirus vector, ChAd3. One study showed that the vaccination of nonhuman primates with a single dose of 10^10^ recombinant adenovirus particles expressing the glycoproteins of *Z. ebolavirus* conferred complete protection from a lethal challenge with *Z. ebolavirus*, whereas a lower vaccine dosage protected most infected animals (Stanely et al., 2014). Given the success of this vaccine platform in nonhuman primates, ChAd3 has now entered a Phase I clinical trial to test its safety, tolerability, and immunogenicity in human volunteers.

In addition to these and other experimental vaccine platforms, monoclonal antibody therapy has been developed for therapeutic use. In particular, a cocktail of three monoclonal antibodies against the *Z. ebolavirus* glycoprotein, called ZMapp (developed by Mapp Biopharmaceutical Inc.), provided complete protection in infected nonhuman primates that were already showing signs of Ebola virus disease (i.e., at five days post-challenge) (Qiu et al., 2014). This success in nonhuman primates prompted the US Food and Drug Administration to approve ZMapp for compassionate use in patients infected with Ebola virus during the current outbreak. Although five patients treated with ZMapp survived their Ebola virus infection, at least two individuals have died from the infection despite receiving the antibody cocktail. Hence, the effectiveness of ZMapp against Ebola virus infection in humans is uncertain and further testing is needed. The antibodies contained in the ZMapp cocktail are produced in tobacco plants, and current stocks of ZMapp have been exhausted.

The development of antiviral drugs against Ebola virus has also progressed. The nucleoside analogs BCX4430 and T-705 (favipiravir) inhibit viral RNA synthesis with a minimal effect on cellular RNA synthesis (Warren et al., 2014, Oestereich et al., 2014). Studies have demonstrated the efficacy of T-705 against Ebola virus in a rodent model (Oestereich et al., 2014), and of BXC4430 against Marburg virus in a nonhuman primate model (Warren et al., 2014). Moreover, both compounds also inhibit other medically important, human RNA viruses (Warren et al., 2014, Oestereich et al., 2014). In particular, T-705 is approved in Japan to combat pandemic influenza. In addition, small molecule therapeutics that include lipid nanoparticle/small interfering RNA technology have been successful in treatment against Ebola virus in nonhuman primates (Geisbert et al., 2010) and have entered clinical trials. Hence, these compounds hold promise as candidates to treat or prevent Ebola infection.

Since the first Ebola outbreak, we have furthered our understanding of this virus and its life cycle, and advances have been made in the development of potential prophylactic and therapeutic treatments. However, we are not there yet, and more basic and translational research is needed. But limited funding has slowed the progress. Additional funding for basic research is essential to identify the virus reservoir, determine the pathogenesis, and identify viral and host targets for antiviral drug discovery. Similarly, more funding for translational research is needed to test novel vaccines and antiviral drugs in nonhuman primates (the gold-standard animal model in Ebola virus research) and to test promising candidates in clinical trials.

The current outbreak is a glaring wake-up call of the devastation the Ebola virus can inflict on the human population. This outbreak has exposed the world's lack of preparedness to respond to and control such infectious disease outbreaks. Our society has concerned itself with the potential of biological terrorist attacks or laboratory-acquired outbreaks; while such scenarios cannot be dismissed, this Ebola virus outbreak makes plain that the true threat of infectious diseases comes from nature. Funding and research (including gain-of-function experiments) must be stable and steady, not dictated by the world's attention, to ensure that we are ready for the next crisis.

## Figures and Tables

**Fig. 1:**
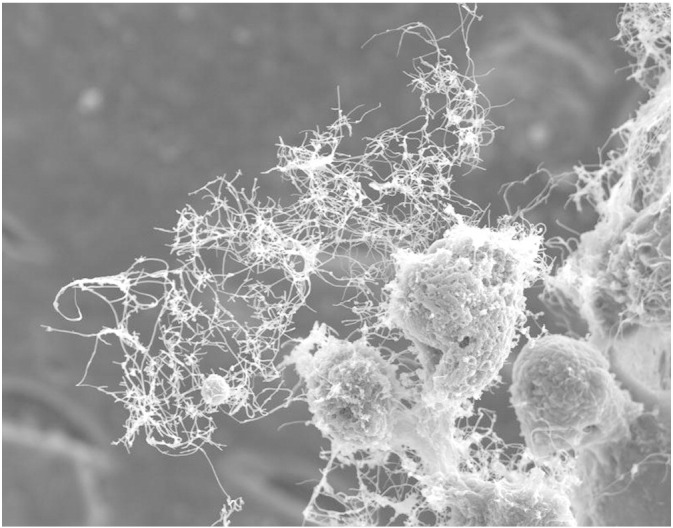
Scanning electron micrograph of cells infected with Ebola virus. Numerous filamentous virions can be seen being released from the cells. Courtesy of Dr. Takeshi Noda, International Research Center for Infectious Diseases, Institute of Medicine Science, University of Tokyo.
